# Composition of carotid plaques differs between Chinese and US patients: a histology study

**DOI:** 10.1186/s41016-025-00408-4

**Published:** 2025-10-09

**Authors:** Jingli Cao, Marina Ferguson, Jie Sun, Mi Shen, Randy Small, Daniel S. Hippe, Xihai Zhao, Dong Zhang, Hiroko Watase, Chun Yuan, Peiyi Gao, James Kevin DeMarco, Roberto F. Nicosia, Yajie Wang, Haowen Li, Zirui Li, Yi Wang, Ted Kohler, Thomas Hatsukami, Binbin Sui

**Affiliations:** 1 China National Clinical Research Center for Neurological Diseases, Beijing Tiantan Hospital, Capital Medical University, Beijing, 100070 China; 2 Vascular Imaging Laboratory, Department of Radiology, University of Washington, Seattle, WA 98195 USA; 3 Department of Radiology, Beijing Tiantan Hospital, Capital Medical University, Beijing, 100070 China; 4 Department of Pathology, University of Washington, Seattle, WA 98195 USA; 5 Clinical Biostatistics, Clinical Research Division, Fred Hutchinson Cancer Center, Seattle, WA USA; 6 Department of Biomedical Engineering, Center for Biomedical Imaging Research, School of Medicine, Tsinghua University, Beijing, 100084 China; 7 Department of Neurosurgery, Beijing Tiantan Hospital, Beijing Neurosurgical Institute, Capital Medical University, Beijing, 100070 China; 8 Department of Surgery, University of Washington, Seattle, WA 98195 USA; 9 The Department of Radiology, Walter Reed National Military Medical Center, Uniformed Services University of Health Sciences, Bethesda, MD 20889 USA; 10 Department of Laboratory Medicine and Pathology, University of Washington, Seattle, 98195 USA; 11 Department of Clinical Laboratory, Beijing Ditan Hospital, Capital Medical University, Beijing, 100015 China; 12 Tiantan Neuroimaging Center for Excellence, Beijing Tiantan Hospital, Beijing Neurosurgical Institute, Beijing, 100070 China

**Keywords:** Carotid artery, Atherosclerotic plaques, Histology, Carotid endarterectomy

## Abstract

**Background:**

The clinical manifestations of cerebrovascular disease are known to differ between the Chinese and United States (US) populations as do the plaque features on imaging.

**Objectives:**

The aim of this study was to investigate and compare the histological features of excised carotid plaques from Chinese and US patients.

**Methods:**

Carotid endarterectomy specimens collected from two prospective studies were included. The entire plaque was serially sectioned (10-µm thickness) at 0.5–1 mm intervals. Hematoxylin and eosin staining and Mallory’s trichrome staining were performed. The morphology and components of the plaques were measured and compared between the two groups.

**Results:**

A total of 1152 histological sections from 75 Chinese patients and 1843 sections from 111 US patients were analyzed. The Chinese group had significantly smaller minimum lumen diameters (median: 1.1 vs. 1.3 mm, *p* = 0.046) and a larger percent wall volume (median: 74% vs. 70%, *p* = 0.018) than the US group. After adjusting for confounding factors, carotid plaques in the Chinese population had larger lipid pools (*β* = 10.0%, 95% *CI*: 4.9 to 15.9%), more recent intraplaque hemorrhage (IPH; *β* = 8.4%, 95% *CI*: 4.5 to 12.7%), less late IPH (*β* = − 8.2%, 95% *CI*: − 11.3 to − 5.4), and fewer fibrous cap disruptions (45% vs. 67%, *p* = 0.061). Chinese plaques were more homogeneous and had a higher percentage of plaques with features of xanthomas than did US plaques (20% vs 2.7%, *p* < 0.001).

**Conclusions:**

The histology of Chinese plaques differs significantly from that of U.S. plaques, suggesting substantial differences in the pathophysiology of atherosclerotic cerebrovascular disease between Chinese and North American populations, which indicates a need for a different management approach.

**Graphical Abstract:**

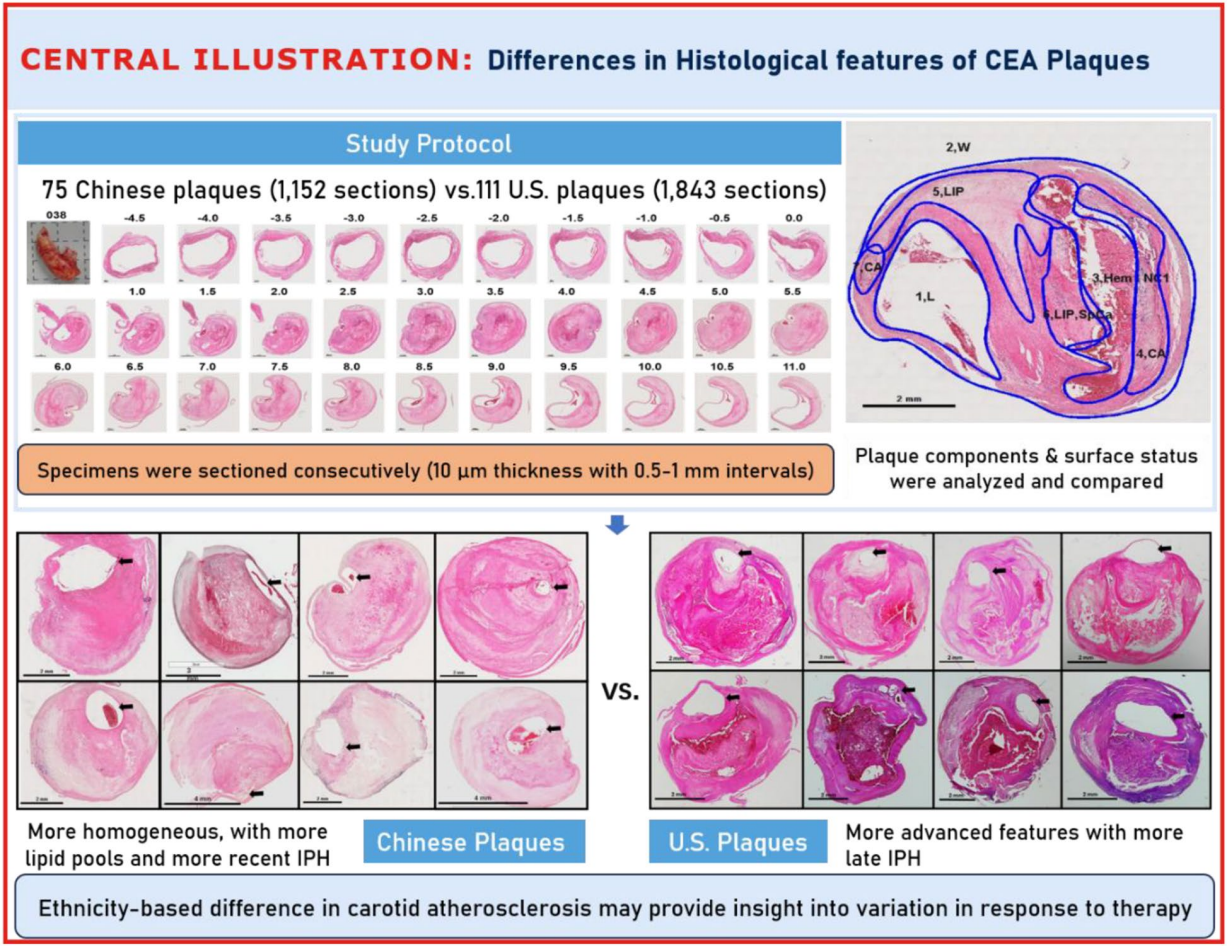

**Supplementary Information:**

The online version contains supplementary material available at 10.1186/s41016-025-00408-4.

## Background

Stroke is one of the leading causes of death and serious long-term disability in China and the USA [1–3]. While these populations share the burden of this potentially preventable disease, there appear to be important differences in the pattern of cerebrovascular disease between China and the USA. When considering atherosclerosis of the large arteries, extracranial disease is more prominent in Caucasian than in Asian patients. Conversely, there is up to a fivefold higher prevalence of intracranial plaque amongst Chinese patients compared to Caucasians [4, 5]. There is also a difference in the distribution of culprit lesions, which are responsible for ischemic events associated with plaque rupture. Intracranial plaques with culprit lesion features are seen three times more frequently in China than in the USA [6]. Our previous pilot studies using magnetic resonance imaging (MRI) also found striking differences in the location and characteristics of potential culprit plaques between two populations on two different continents [7, 8].

Ethnicity-based characteristics and differences have been highlighted in previous studies on coronary, intracranial artery and carotid intima-media thickness [9–11]. Difference in the characteristics of atherosclerotic disease may be linked to different approaches to treatment and prevention. However, the histological differences in carotid advanced atherosclerotic plaques have not been extensively investigated.

Herein, we report a study comparing the pathology of excised carotid plaques from patients undergoing carotid endarterectomy (CEA) in the USA and China. We quantified and compared plaque components of endarterectomy specimens from the University of Washington (UW) and from the Beijing Tiantan Hospital (BTH). Plaques were removed intact to preserve luminal surface morphology and were comprehensively analyzed in toto.

## Methods

### Study population

CEA specimens were collected from 75 BTH patients in the Culprit Plaque in Acute Cerebral infarction (CPAC) study between 2014 and 2018 and from 111 patients in the UW study between 1997 and 2008. Less than 3% of the subjects enrolled in the US cohort have been Asian, which reflects the prevalence of Asian-American patients undergoing CEA in the UW study. The indications for operation in both countries followed the guidelines of the Asymptomatic Carotid Atherosclerosis Study [12], the European Carotid Surgery Trial [13], and the North American Symptomatic Carotid Endarterectomy Trial [14]. Clinical risk factors and medication use (statins, antiplatelet agents, and anticoagulant agents) were recorded through retrospective review of the medical chart. Approval was obtained from the local Institutional Research Boards at each institution (UW STUDY 00003635, BTW KY2014-004–01), and the procedures followed were in accordance with institutional guidelines. All subjects gave written informed consent.

### Surgery and specimen processing

All specimens were removed without disruption by incising the carotid longitudinally through the adventitia and outer media but not into the plaque. Thus, the plaque wall and luminal surface were never violated (Supplement Fig. 1, right). The thickness of the artery wall left behind was less than 1 mm.

**Fig. 1:**
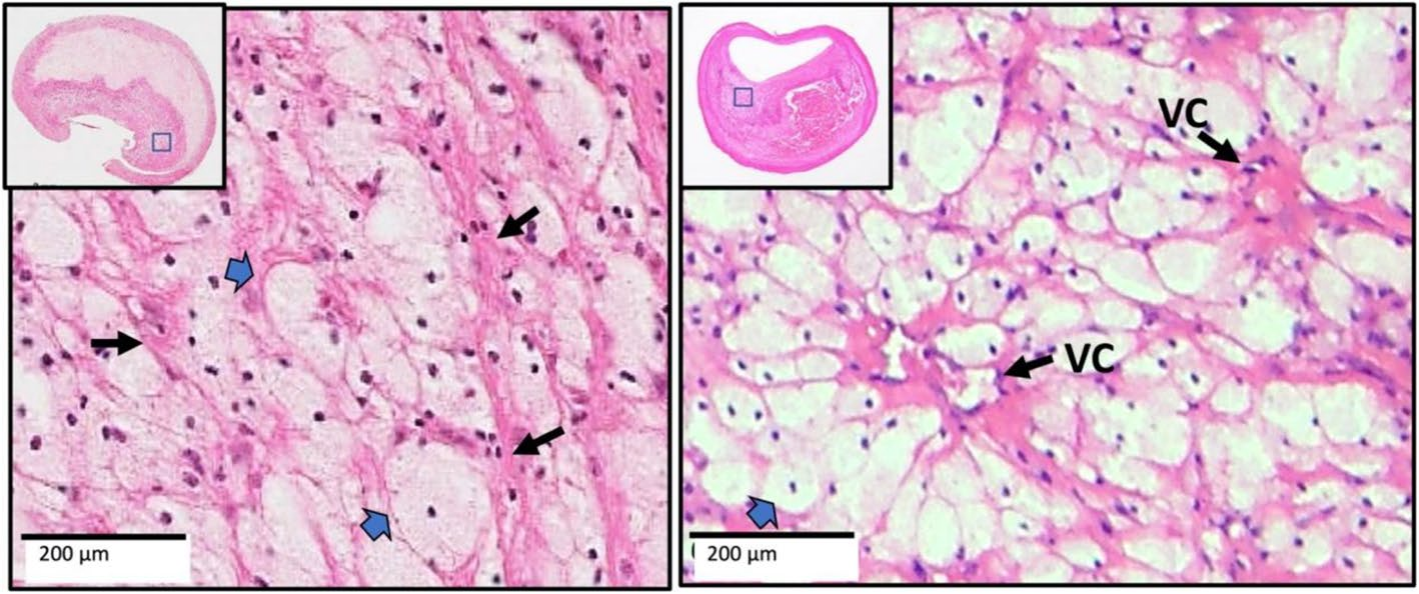
Two xanthomas with similar characteristics are shown from China (left) and from the US (right). Narrow arrows point to the delicate interstitial collagen fibrils, and block arrows point to foamy macrophages. The arrangement of macrophages around vascular channels (VC) is the characteristic of xanthomas (H&E)

Protocols for specimen processing were standardized between the two centers. Plaques were processed by two histologists: Marina Ferguson, MSF, with over 20 years of experience in plaque histological analysis and Jingli Cao, JLC, with over 10 years of experience. First, the plaques were gently flushed with Ringer’s lactate and placed in 10% neutral buffered formalin for a minimum of 24 h. Specimens were decalcified with RapidCal·Immuno™ (Zhongshan Golden Bridge Biotechnology, Beijing, China) in China and 10% formic acid in the USA. Specimens were embedded en bloc in paraffin and sectioned (10-µm thickness) at 1 mm intervals in the common and 0.5 mm intervals in the internal carotid artery. The entire specimen was processed and analyzed. The sections were stained with hematoxylin and eosin (H&E) and with Mallory’s trichrome.

### Plaque morphometry analysis

Images for the Chinese specimens were captured on Aperio ImageScope, Pathology Viewing System v12.0.1.5027 (Leica Biosystems, Wetzlar, Germany), and Ventana Image Viewer v.3.1.3 (Ventana Medical Systems, Tucson, AZ, USA). US images were captured with a Nikon COOLPIX camera. Images of H&E sections at 1 × magnification were transferred to MATLAB R2014a (MathWorks, Natick, MA, USA) for digitizing with QVAS (Quantitative Vascular Analysis System, 2004 Histology Version, Vascular Imaging Laboratory, University of Washington, Seattle, USA) [15]. During digitizing, reference was made to the original glass slides using 200 × magnification to classify plaque features based on detailed analysis of their components.

All histopathological images were analyzed by a single reviewer (Marina Ferguson, MSF). For each cross-sectional location, the total area of the specimen was measured by outlining its outer border and the lumen area by the inner perimeter of the wall. The wall area was calculated as the difference between the total area and the lumen area. Total vessel volume and wall volume were calculated by summing the corresponding areas and then multiplying by the spacing interval (0.5 or 1.0 mm). Overall plaque burden was defined as 100% × wall volume/total vessel volume. The percent narrowing at each section was calculated as 100% × (1 – lumen area/total vessel area), and the maximum value was recorded as the stenosis degree.

### Classification and analysis of plaque components and surface status

Histologic features of endarterectomy specimens from the two groups were identified, classified, and outlined. For each component, the corresponding percentages of component volume were calculated as: 100% × component volume/wall volume. Plaque compositional features were classified according to the histopathologic system described by Stary et al. from the Committee on Vascular Lesions of the Council of Atherosclerosis, American Heart Association [15]. These were as follows (Supplement Fig. 2):

1. *Lipid pools* are early lesions of morphologically distinct spaces composed of phospholipids, cholesterol monohydrate, and cholesterol esters. Due to the extraction of fats from the plaque by alcohol during histological processing, lipid pools are visualized by H&E as acellular spaces that may contain small cholesterol clefts and faintly staining, grainy, amorphous material [15]. Lipid pools do not stain with Mallory’s trichrome, which stains fibrin, or anti-glycophorin A, which attaches to the sialoglycoprotein glycophorin A on the red cell membrane [16].
2. *Necrotic core *is a region filled with necrotic debris and which displace fibrous tissue. The core may have a well-defined edge populated by an inflammatory response. It may also contain lipid, cholesterol clefts, red blood cell fragments, collagen fragments, and calcifications [17]. Regions containing a necrotic core were distinguished from areas of dense, collagen-rich fibrous intimal tissue and regions with loose connective tissue matrix.
3. *Recent intraplaque hemorrhage *is a mixture of intact and degenerating red blood cells, hemosiderin-laden macrophages, giant cells, and cholesterol crystals. Peripheral neoangiogenesis and speckled calcifications also may be present [18]. Red blood cells are eosinophilic and lose some of this tinctorial quality when they degenerate and become de-hemoglobinized. Therefore, hemorrhagic areas show various degrees of pink staining on hematoxylin and eosin. Mallory’s trichrome results in a red color for fibrin, fibrin products, and red blood cells, whereas extracellular matrix components stain blue. When hemorrhage occurs outside a necrotic core, it is composed of intact and decomposing red blood cells, fibrin and fibrin products, and a few inflammatory cells.
4. *Late intraplaque hemorrhage *is an amorphous, faintly eosinophilic material which may contain small and large cholesterol clefts along with cellular debris, hemosiderin, and a variety of calcifications [18]. The hemorrhagic nature of this material, which no longer contains intact red blood cells, is confirmed by staining for the red blood cell membrane protein glycophorin A which provides specific evidence of prior bleeding in these areas [19].
5. *Calcification* is composed of hydroxyapatite crystals ranging in size from small intracellular granules [20] to large amorphous extracellular deposits [21]. The decalcification process removes enough calcium to permit histological sectioning, but traces of calcium, bound to phosphate or carbonate anions, remain on the periphery of the calcified region giving a characteristic blue outline when stained with hematoxylin.
6. *Loose extracellular matrix* is characterized by interlacing, loosely organized collagen fibrils in an amorphous proteoglycan-rich background which is populated by smooth muscle cells, fibroblasts, and varying numbers of microvessels. A similar type of matrix is found in organizing thrombi or on the luminal surface.
7. *Xanthomas* are distinct accumulations of lipid laden macrophages (foam cells) separated by richly vascularized collagenous matrix and arranged in a lobular pattern. A focal mixed inflammatory infiltrate is frequently present. Cholesterol clefts are sometimes noted. Xanthomas do not have the lipid-rich necrotic center that is typically present in the fibroatheromas where foam cells are haphazardly arranged and randomly admixed with cholesterol clefts and cell debris. Xanthomas demonstrate delicate interstitial collagen and are similar to those found in patients with lipid disorders (Fig. 1) [22, 23].

**Fig. 2:**
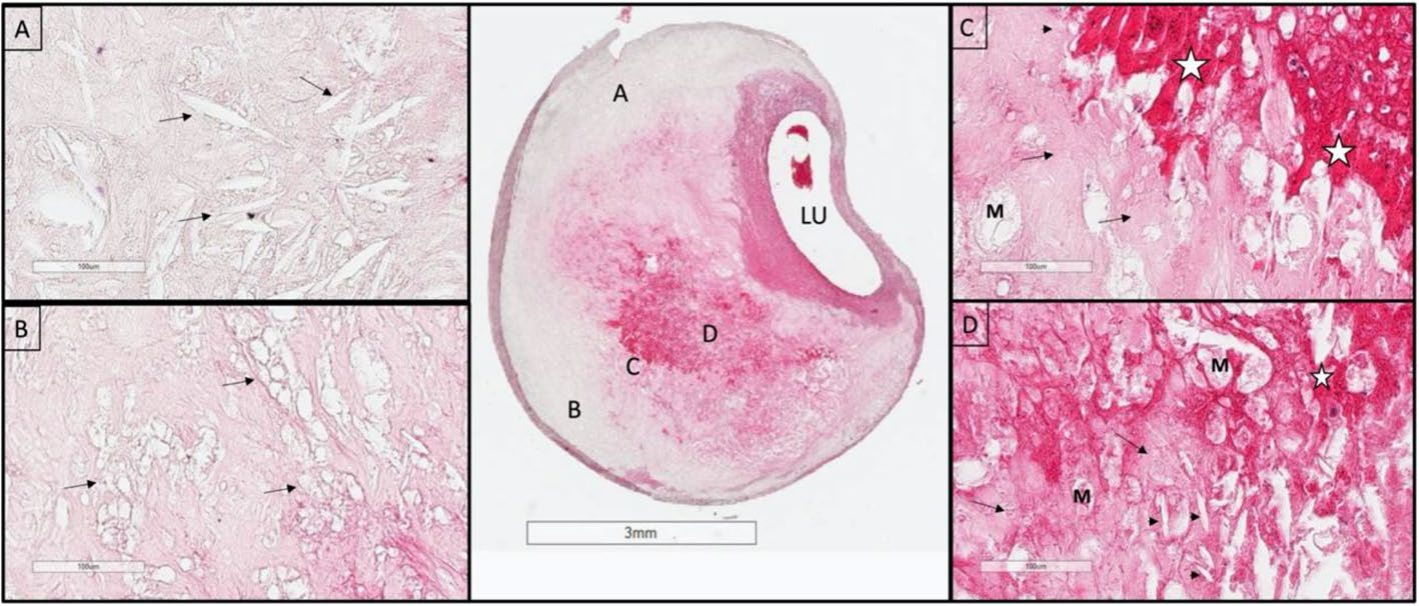
An example of the homogeneity of a Chinese plaque. Large deposits of minimally staining lipid and necrosis are predominant. **A** Shows cholesterol clefts (arrows) in an acellular necrotic deposit. **B** Shows clusters of dead macrophages (arrows) suspended in a simple matrix. **C** Shows the junction of matrix (arrows) and intraplaque hemorrhage (IPH) (star). **D** Illustrates the mixture of IPH (star), matrix (arrows), macrophages (M), and cholesterol clefts (arrow heads). LU indicates the lumen (H&E)

Plaque surface disruptions were defined and characterized as follows:

1. *Fibrous cap disruption*: A disruption of the fibrous tissue between the necrotic core and the lumen.
2. *Disruption with mural thrombus*: A lumen surface lacking an endothelial cell covering and with an attached thrombus composed of blood components such as platelets, fibrin, and red and white blood cells.
3. *Disruption with juxtaluminal calcification*: Calcifications protruding into the lumen from a luminal plaque disruption.

### Data analysis

Continuous variables were summarized as median (inter-quartile range [IQR]), and categorical variables were summarized as count (percentage). The Wilcoxon rank-sum test and Fisher’s exact test were used to compare clinical and plaque characteristics between two groups. Linear regression was used to compare each plaque composition variable (% volume, outcome variable) between groups (Chinese vs. US cohort or medication exposure), adjusted for recent carotid symptoms, carotid stenosis, sex, age, other cardiovascular risk factors and medications. Logistic regression was used to compare each luminal surface status variable (outcome variable) between groups, adjusted for the same covariates. The adjusted mean difference in plaque component volume between groups was summarized using the linear regression coefficient (*β*), and the adjusted difference in surface status between groups was summarized using the adjusted odds ratio (OR) from the logistic regression model. The nonparametric bootstrap was used to calculate 95% confidence intervals (CIs) for the comparisons of plaque composition between groups based on linear regression models to avoid making distributional assumptions.

Associations of plaque composition and surface conditions with recent carotid symptoms were assessed using logistic regression models with symptoms as the outcome variable. The square root transformation was applied to each composition volume variable to reduce skewness before inclusion in the corresponding model. Each model was adjusted for cohort (Chinese vs. USA), carotid stenosis, sex, age, and the other factors listed in Table 1. Differences in the associations with symptoms between Chinese and US groups (summarized using the OR) were tested by adding a cohort-by-plaque-feature interaction term to each model. All statistical calculations were conducted with the statistical computing language R (version 4.0.3; R Foundation for Statistical Computing, Vienna, Austria).

**Table 1:** Demographic data and clinical characteristics of the patients

	Cohort*				
CVD risk factors	Chinese group (*N* = 75)		US group (*N* = 111)	*p*-value†	
Male sex	58 (77.3)		101 (91.0)	0.011	
Age, years	63 (58–67)		70 (62–76)	< 0.001	
Hypertension	50 (66.7)		91 (82.0)	0.023	
Hyperlipidemia‡	29 (39.7)		90 (85.7)	< 0.001	
Diabetes	24 (32.0)		28 (25.2)	0.32	
Current smoker	27 (36.0)		41 (36.9)	> 0.99	
CVD history					
Symptomatic carotid artery	22 (29.3)		37 (33.3)	0.63	
Coronary artery disease	10 (13.3)		50 (45.0)	< 0.001	
Medications					
Statins	24 (32.0)		74 (66.7)	< 0.001	
Antiplatelets or anticoagulants	15 (20.0)		75 (67.6)	< 0.001	

^*^Values no. (%) or median (range)

^†^Fisher’s exact test or the Wilcoxon rank-sum test

^‡^Eight subjects were missing a history of hyperlipidemia (six Chinese and two US)

## Results

### Study population and vascular risk factors

A total of 1152 sections from 75 Chinese plaques and 1843 sections from 111 US plaques were analyzed. The number of sections per plaque were similar (Chinese group: median 15, range 5–28; US group: median 17, range 6–34). The demographic data of the Chinese and US groups are listed in Table 1. The Chinese group was younger with more females and less hypertension, hyperlipidemia, and coronary artery disease (all *p* < 0.05). No significant difference was found in the proportion of symptomatic carotids between the two cohorts (29% vs. 33%, *p* = 0.63). Significantly fewer Chinese patients were taking statins or anti-thrombotics prior to admission (all *p* < 0.001).

### Plaque morphology

The maximum outer wall diameters were similar between the two groups (median: 9.2 vs. 9.2 mm, *p* = 0.82), as were the plaque lengths (median: 14 vs. 15 mm, *p* = 0.66) (Table 2). The Chinese group had significantly smaller minimum lumen diameters than the US group (median: 1.1 vs. 1.3 mm, *p* = 0.046), which translated into a small though statistically significantly more severe stenosis in the Chinese plaques (median [IQR]: 97% [93–99%] vs. 96% [91–98%], *p* = 0.005). The Chinese plaques also had a larger overall plaque burden than the US plaques in terms of percent wall volume (median: 74% vs. 70%, *p* = 0.018).

**Table 2:** Comparison of plaque morphology between two groups

	Cohort*		
Morphology	Chinese groups (*N* = 75)	US group (*N* = 111)	*p*-value†
Plaque length, mm	14 (10–17)	15 (11–18)	0.66
Maximum outer wall diameter, mm	9.2 (8.2–10.0)	9.2 (8.3–10.0)	0.82
Minimum lumen diameter, mm	1.1 (0.6–1.6)	1.3 (0.9–1.9)	0.046
Stenosis, %	97 (93–99)	96 (91–98)	0.005
Percent wall volume, %	74 (66–81)	70 (64–76)	0.018

^*^Values no. (%) or median (inter-quartile range)

^†^Fisher’s exact test or the Wilcoxon rank-sum test

### Plaque composition

**Fig. 3:**
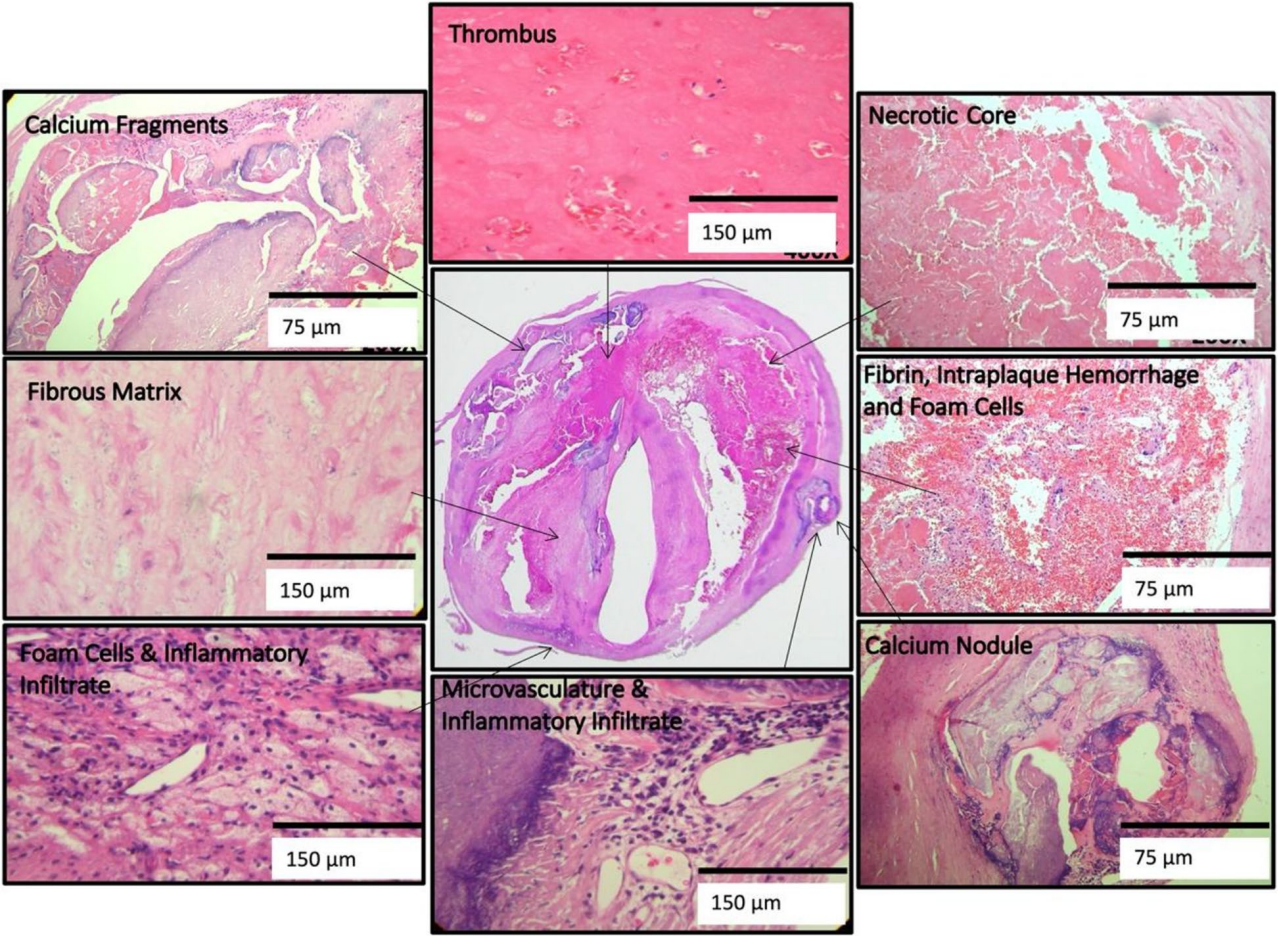
High-power illustrations of common components found in the heterogeneous US plaques (clockwise): thrombus; necrotic core; fibrin, intraplaque hemorrhage, and foam cells; calcium nodule; microvasculature and inflammatory infiltrate; foam cell aggregates and inflammation; and fibrous matrix and calcium fragments (H&E)

**Fig. 4:**
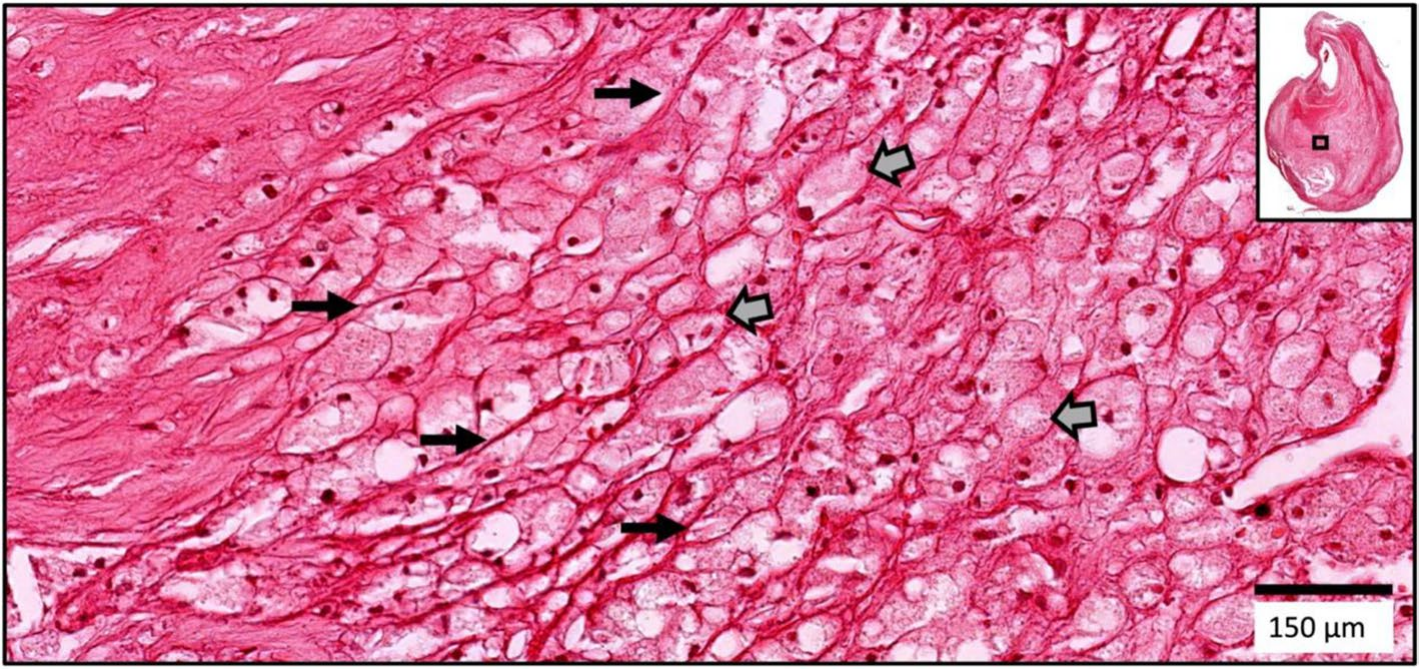
A fibro-lipid Chinese plaque. Large foam cells (block arrows) are sequestered along extracellular matrix fibers (long arrows) in this fibro-lipid Chinese plaque (H&E)

**Fig. 5:**
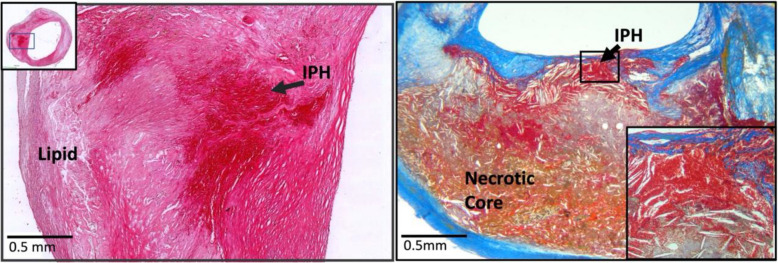
Recent intraplaque hemorrhage (IPH) into the fibrous matrix of a Chinese plaque (left) (H&E). Recent intraplaque hemorrhage into a necrotic core of a US plaque (right) (Mallory’s trichrome)

When examined at low magnification, Chinese plaques had a more homogeneous morphology (Fig. 2) than US plaques, which were frequently complex due to a greater admixture of different components (Fig. 3). Plaques from the US cohort were composed of lesions typical of type VI atheroma where most IPH spills into a necrotic core. Features that contributed to the complexity of the US plaques included broader areas of necrosis, calcifications, thrombosis, and hemorrhage. Chinese specimens contained large lipid deposits, necrotic debris, cholesterol clefts, and uniform extracellular matrix. The plaques had prominent fibro-lipid accumulations with foam cells sequestered along fibers of loose extracellular matrix (Fig. 4). In Chinese plaques, old IPH was found in necrotic cores, but additional recent IPH were often found in fibrous matrix or adjacent to xanthomas or inflammatory infiltrate (Fig. 5). Chinese plaques more frequently had lipid in foam cells with the pattern of xanthomas (15/75, 20%) than in Western plaques (3/111, 2.7% *p* < 0.001). All components except late hemorrhage were significantly correlated with percent stenosis, and all features except calcification were significantly associated with percent wall volume (Supplement Table 1).

Table 3 lists the difference in plaque composition (% volume of components) between Chinese and US groups. Prior to adjustment for possible confounders, the Chinese plaques were comprised of larger lipid pools (median: 11.6% vs. 2.9%, *p* < 0.001) but smaller necrotic cores than the US plaques (median: 8.5% vs. 15.0%, *p* < 0.001). The US plaques contained larger IPH overall than the Chinese plaques (median: 17.7%, vs. 9.3%, *p* = 0.011). The Chinese plaques contained more recent IPH (median: 5.2% vs. 1.5%, *p* = 0.002) and less late IPH (median: 2.0% vs. 10.2%, *p* < 0.001) than the US plaques. Volumes of calcification (median: 6.2% vs. 7.6%, *p* = 0.63) and loose matrix (median: 1.2% vs. 1.4%, *p* = 0.96) were similar in the two cohorts.

**Table 3:** Comparison of plaque composition and surface status

	Cohort*			Adjusted comparisons†		
Composition volume%	Chinese group (*N* = 75)	US group (*N* = 111)	*p*-value‡	Adj β	(95% CI)	*p*-value
Lipid pool, %	11.6 (4.8–27.2)	2.9 (0.7–9.1)	< 0.001	10.0%	(4.9, 15.9)	< 0.001
Necrotic core, %	8.5 (2.7–19.3)	15.0 (4.1–26.1)	0.031	0.3%	(− 4.5, 4.9)	0.94
IPH, %	9.3 (4.9–21.5)	17.7 (6.9–28.0)	0.011	0.2%	(− 4.5, 4.7)	0.94
Recent IPH, %	5.2 (0.9–17.4)	1.5 (0.0–8.1)	0.002	8.4%	(4.5, 12.7)	< 0.001
Late IPH, %	2.0 (0.5–5.3)	10.2 (2.4–21.9)	< 0.001	− 8.2%	(− 11.3, − 5.4)	< 0.001
Calcification, %	6.2 (2.5–15.9)	7.6 (2.0–19.7)	0.63	3.0%	(− 3.1, 9.2)	0.34
Loose matrix, %	1.2 (0.0–6.1)	1.4 (0.3–3.8)	0.96	1.6%	(− 0.6, 4.1)	0.19
				Adjusted comparisons†		
Surface status				Adj OR	(95% CI)	*p*-value
Fibrous cap rupture	34 (45.3)	74 (66.7)	0.004	0.43	(0.18, 1.04)	0.061
Mural thrombus	10 (13.3)	15 (13.5)	> 0.99	0.45	(0.11, 1.75)	0.25
Juxtaluminal calcification	30 (40.0)	65 (58.6)	0.017	0.97	(0.42, 2.27)	0.95

*OR* odds ratio for surface status, *CI* confidence interval, *IPH* intraplaque hemorrhage

^*^Values no. (%) or median (inter-quartile range)

^†^The multivariable analysis was based on 178 of 186 patients (96%) with complete risk factor data. The regression coefficient (*β*) or OR corresponds to the mean difference in composition or difference in risk of surface condition between plaques from the Chinese and US cohorts after adjusting for symptom status, stenosis, sex, age, and the following risk factors and medications: hypertension, hyperlipidemia, diabetes, smoking status, coronary artery disease, statins, and use of antiplatelets/anticoagulants

^‡^Fisher’s exact test or the Wilcoxon rank-sum test comparing cohorts without covariate adjustment

After adjustment for symptom status, stenosis, sex, age, other risk factors, and medications, Chinese plaques still had significantly greater volumes of lipid pools (adjusted *β*: 10.0% [95% *CI*: 4.9, 15.9%], *p* < 0.001) and recent hemorrhage (adjusted *β*: 8.4% [95% *CI*: 4.5–12.7%], *p* < 0.001) and smaller volumes of late hemorrhage than US plaques (adjusted *β*: − 8.2% [95% *CI*: − 11.3, − 5.4%], *p* < 0.001) (Table 3). However, the adjusted differences in necrotic core (adjusted *β*: 0.3% [95% *CI*: − 4.5, 4.9%], *p* = 0.94) and total hemorrhage (adjusted *β*: 0.2% [95% *CI*: − 4.5, 4.7%], *p* = 0.94) were no longer statistically significant.

### Lumen surface status

Surface disruptions due to fibrous cap rupture were less common in Chinese specimens than in those in the US cohort (45.3% vs. 66.7%, *p* = 0.004) as were disruptions with juxtaluminal calcification (40.0% vs 58.6%, *p* = 0.017). Frequencies of disruptions associated with mural thrombus were similar in the two cohorts (13.3% vs. 13.5%, *p* > 0.99).

After adjustment for symptom status, stenosis, sex, age, the risk factors, and medications, there was still a trend towards a lower prevalence of fibrous cap rupture in Chinese plaques than US plaques (adjusted *OR*: 0.43 [95% *CI*: 0.18, 1.04], *p* = 0.061). The prevalence of disruption associated with mural thrombus (*p* = 0.25) or juxtaluminal calcification (*p* = 0.95) was not significantly different between Chinese and US plaques after adjustment.

### Associations of plaque features with medications and symptoms

After adjusting for cohort (Chinese vs. US), symptom status, stenosis, and other characteristics, there were no significant associations between statin use and either plaque composition or surface status (Table 4). IPH volume was significantly larger in plaques from patients with exposure to antiplatelet or anticoagulant agents than in those without this exposure (adjusted *β*: 4.1% [95% *CI*: 0.0, 8.3%], *p* = 0.048). This difference in total IPH appeared mainly to be due to differences in recent IPH (adjusted *β*: 3.0%, *p* = 0.059) rather than late IPH (adjusted *β*: 1.1%, *p* = 0.47). There was also a trend towards an association between antiplatelet or anticoagulant use and juxtaluminal calcification (adjusted *OR*: 1.92, *p* = 0.077).

**Table 4:** Associations between medications, plaque composition, and surface status

	Associations with statins*			Associations with antiplatelets/anticoagulants†		
Composition volume%	Adj. β‡	(95% CI)	*p*-value	Adj. β‡	(95% *CI*)	*p*-value
Lipid pool, %	2.4	(− 2.4, 6.8)	0.31	− 1.4	(− 5.6, 2.9)	0.50
Necrotic core, %	0.9	(− 4.9, 6.3)	0.76	3.2	(− 1.0, 7.5)	0.14
IPH, %	0.9	(− 4.3, 5.8)	0.72	4.1	(0.0, 8.3)	0.048
Recent IPH, %	2.3	(− 1.9, 6.4)	0.27	3.0	(− 0.1, 6.3)	0.059
Late IPH, %	− 1.4	(− 5.8, 2.7)	0.50	1.1	(− 1.8, 4.1)	0.47
Calcification, %	− 0.7	(− 7.0, 5.6)	0.83	− 0.8	(− 5.3, 3.8)	0.71
	Associations with statins*			Associations with antiplatelets/anticoagulants†		
Surface status	Adj. OR‡	(95% CI)	*p*-value	Adj. OR‡	(95% CI)	*p*-value
Fibrous cap rupture	0.62	(0.20, 1.85)	0.39	1.30	(0.61, 2.78)	0.49
Mural thrombus	0.56	(0.14, 2.26)	0.41	0.85	(0.26, 2.75)	0.78
Juxtaluminal calcification	0.80	(0.28, 2.28)	0.68	1.92	(0.93, 3.97)	0.077

*OR* odds ratio for surface status, *CI* confidence interval, *IPH* intraplaque hemorrhage

^*^The comparison between those with and without statin use are adjusted for cohort (Chinese vs. US), carotid stenosis, carotid symptoms, sex, age, and the following clinical characteristics: hypertension, hyperlipidemia, diabetes, smoking status, coronary artery disease, and use of antiplatelets/anticoagulants

^†^The comparison between those with and without antiplatelets/anticoagulants use is adjusted for cohort (Chinese vs. US), carotid stenosis, carotid symptoms, sex, age, and the following clinical characteristics: hypertension, hyperlipidemia, diabetes, smoking status, carotid symptoms, coronary artery disease, and statin use

^‡^The multivariable analysis was based on 178 of 186 patients (96%) with complete risk factor data. The regression coefficient (*β*) or OR corresponds to the mean difference in composition or difference in risk of surface condition between plaques with and without exposure to the corresponding medication after multivariable adjustments

After adjusting for cohort, stenosis, and other characteristics, there were no statistically significant associations between any plaque component or surface condition and carotid symptoms (Table 5). However, there were trends towards larger necrotic core (adjusted *OR*: 1.38 per 1-SD increase, *p* = 0.096) and IPH (adjusted *OR*: 1.49 per 1-SD increase, *p* = 0.052) being associated with symptoms. Recent IPH (adjusted *OR*: 1.33, *p* = 0.13) and late IPH (adjusted *OR*: 1.31, *p* = 0.18) had similar contributions. No associations with symptoms were significantly different between Chinese and US plaques (*p* > 0.14, Table 5).

**Table 5:** Associations of plaque composition and surface status with carotid symptoms

	Associations with symptoms*			Difference between Chinese and US
% composition volume	Adj. OR†	(95% CI)	*p*-value	*p*-value‡
Lipid pool, %	1.16	(0.80, 1.69)	0.42	0.17
Necrotic core, %	1.38	(0.94, 2.03)	0.096	0.94
IPH, %	1.49	(1.00, 2.22)	0.052	0.77
Recent IPH, %	1.33	(0.92, 1.93)	0.13	0.49
Late IPH, %	1.31	(0.89, 1.94)	0.18	0.46
Calcification, %	1.10	(0.79, 1.54)	0.57	0.39
Surface status				
Fibrous cap rupture	1.08	(0.54, 2.16)	0.83	0.63
Mural thrombus	1.55	(0.60, 4.03)	0.37	0.14
Juxtaluminal calcification	1.32	(0.66, 2.62)	0.43	0.66

*OR* odds ratio for association with symptoms, *CI* confidence interval, *IPH* intraplaque hemorrhage

^*^The multivariable analysis was based on 178 of 186 patients (96%) with complete risk factor data

^†^The OR for each composition variable is scaled to correspond to change per 1-SD increase after square root transformation to reduce skewness; the ORs are adjusted for cohort (Chinese vs. US), carotid stenosis, sex, age, and the following clinical characteristics: hypertension, hyperlipidemia, diabetes, smoking status, coronary artery disease, statin use, and use of antiplatelets/anticoagulants

^‡^Wald test of an interaction between cohort (Chinese vs. US) and the corresponding composition or surface status variable

## Discussion

In the current study, the histological features of carotid plaque samples from China and the USA were comprehensively compared, with analysis of the entire length of CEA specimens removed without surgical disruption of the plaque or luminal surface. To our knowledge, this is the first such report incorporating detailed analysis of histological cross-sections obtained every 0.5 to 1.0 mm, thus avoiding bias from review of a smaller number of selected locations within the atheroma.

### Differences in plaque composition

Results demonstrated that there were significant differences in carotid plaque composition between the two groups. Despite more severe stenosis and larger plaque burden, Chinese plaques had fewer features of advanced atherosclerosis and were more homogenous, with significantly more recent IPH and larger accumulations of lipid. Late IPH and a trend toward more fibrous cap rupture, commonly considered as complex manifestations of a later stage of atherosclerosis, were found more commonly in US plaques. These findings suggest that the fundamental pathophysiology of atherosclerosis in Chinese and US cohorts may differ and provides a histological reference for future comparative research, for example, in studies utilizing vessel wall MRI [8]. Such studies, incorporating serial imaging of the carotid atheroma, will be critical to assess differences in response to treatment between groups and perhaps aid in therapeutic decision-making.

In a pilot study using MRI, plaque composition was compared between 20 symptomatic Chinese and 20 US Caucasian patients with > 50% stenosis [7]. The US cohort had a significantly higher prevalence of advanced lesions (AHA type VII) including luminal calcifications with juxtaluminal IPH and mural thrombus. Similar findings were noted in another MRI study of recently symptomatic Chinese and US patients regardless of the degree of stenosis: features of high-risk plaques (IPH or surface disruption) were significantly more frequent in extracranial carotids in the Caucasian cohort (42% vs 16%) [8]. These findings, along with dissimilarities in morphology and distribution noted in prior imaging studies, indicate significant differences in cerebrovascular pathophysiology between Chinese and North American populations, suggesting that a different approach to treatment and prevention may be needed.

### Difference in IPH stage and location

The detailed histological analysis of the specimens in this study identified distinct patterns of IPH in our two cohorts. Over 90% of both US and Chinese plaques in our study had IPH. This high prevalence is consistent with the work of Derksen et al., who found an 81% prevalence of IPH in 794 carotid specimens [18]. However, our histology results revealed that the stage and location of hemorrhage differed in our two cohorts. Recent IPH was present more frequently and in greater volume in Chinese plaques, while older IPH was more common and voluminous in US plaques, suggesting that recurrent IPH over time into advanced plaques is more common in US than in Chinese patients. This finding provides further evidence of the greater complexity of plaques in US group. In US plaques, almost all IPH occurred in the necrotic core, while in Chinese plaques, recent IPH was often found in fibrous matrix or adjacent to xanthomas or inflammatory infiltrate. The location of IPH (within a necrotic core or within fibrous matrix) might be a critical factor in how it impacts vulnerability to plaque rupture and embolization. Thus, further studies are needed to assess the association of recent IPH and its location to risk of embolic disease.

### Lipid accumulation patterns in Chinese plaques

Results from this study revealed that Chinese plaques were more homogeneous with larger lipid accumulations. Further, a histologic feature identified as xanthomas was found much more common in Chinese plaques. The term xanthoma was utilized due to the similar histologic features as in xanthomas found in skin and tendon lesions. Morphologically, these distinct collections of foam cells embedded in a delicate and vascularized stroma resemble cholesterol-rich tendon xanthomas found in patients with familial hypercholesterolemia [24]. This is the first study proposing this term with specific features in carotid plaques, which may be a consequence of most previous investigations being based on Western cohorts. Further studies are needed to ascertain the clinical significance and implications of carotid xanthoma. The differences in composition and complexity found in this study highlights the need for histological studies in populations with greater diversity in ethnic background and geography.

### Plaque composition, clinical risk factors and medications

Our results showed that several clinical risk factors, as well as statin and antithrombotic drug use, were significantly different between the two groups, which may have contributed to differences in the carotid plaques. However, after adjusting for cohort (Chinese vs. US), symptom status, stenosis, and other characteristics, there were no significant associations between statin use and plaque composition. Antiplatelet use was associated with a greater volume of IPH, consistent with prior reports of increased IPH in association with the use of antithrombotic treatment [25]. We found a higher prevalence of recent intraplaque hemorrhage in Chinese plaques, yet paradoxically, antiplatelet or anticoagulant use on admission was much less common in the Chinese cohort. Further MRI studies on clinical and risk factors associated with the development of new or recurrent carotid IPH are ongoing.

### Limitations

First, since the plaque specimen was obtained from patients undergoing CEA, our cohort consisted of patients with significant carotid stenosis, and thus does not provide data on the characteristics of plaques in patients with mild to moderate stenosis. Also, we focused on histologic features that may be localized and quantified by clinical imaging techniques, realizing that more detailed analysis of cell types and local biochemical milieu also play a critical role in establishing plaque vulnerability. Second, the number of plaques with mural thrombus was relatively low (25 total), so analyses of this feature were more exploratory. Lastly, the collection time of the plaques in two groups of this study was approximately one to two decades apart, with the Chinese cohort more contemporary. Some of the differences noted in this study may be related to changes in disease pattern and treatment over time. However, our recent comparison of morphological and compositional characteristics in carotid plaques of two Chinese cohorts at different eras (2002–2005 and 2012–2015) using MRI showed there was no significant difference in IPH during different eras [26], which suggest that the differences in the pattern and stage of IPH between cohorts in this study are not entirely due to difference in time period. The former work also indicated that plaque lipid content was lower amongst the more recent cohort. In this paper, we found there were more lipid pools in Chinese compared to US plaques. Further comparative studies by serial imaging are needed to confirm the results of this study and to determine the natural history and clinical outcome associated with various plaque morphologies.

## Conclusions

In conclusion, Chinese plaques had fewer features of advanced atherosclerosis and were more homogenous with significantly more recent IPH and larger accumulations of lipid than US plaques. These findings demonstrate that atherogenesis may produce fundamentally different lesions in different populations on different continents. Understanding these differences may allow better risk assessment and individualization of treatment in different populations.

### Clinical perspective

#### Clinical competencies

1. Carotid plaque composition was significantly different in these Chinese and US cohorts.
2. Chinese plaques were less complex with larger lipid accumulations and more recent intraplaque hemorrhages and tended to have fewer fibrous cap ruptures. US plaques had more advanced features such as late intraplaque hemorrhage.

#### Translational outlooks

1. These histologic findings together with prior MRI studies suggest a significant difference in the underlying pathophysiology of cerebrovascular disease in Chinese and US populations that may require a different approach to management.
2. Investigations of the population differences in carotid advanced atherosclerosis may provide insight into variations in response to therapy between ethnic groups and help guide strategies for treatment.
3. Further studies on intra- and extracranial atheroma with mild to moderate stenosis, along the follow-up imaging, will provide insight into whether there are any differences in earlier stages of atherosclerosis development and progression between Chinese and US cohorts. List of Abbreviations AS: Atherosclerosis MRI: Magnetic resonance imaging CEA: Carotid endarterectomy LRNC: Lipid-rich necrotic core LPH: Low-pressure hydrocephalus IPH: Intraplaque hemorrhage CI: Confidence interval OR: Odds ratio

## Supplementary Information

Supplementary Material 1. Supplement Fig. 1 Left. Carotid plaque from a standard endarterectomy with disruption of the specimen. Right. Specimen from the modified surgical technique without disruption of the inner surface. Corresponding histologic sections are below.

Supplementary Material 2. Supplement Fig. 2 Carotid plaque components: A) Lipid pool (arrow)(H &E); B) Necrotic core with thick fibrous cap (arrow) (Mallory’s trichrome); C) Recent intraplaque hemorrhage into a necrotic core (star) (Mallory’s trichrome). Late intraplaque hemorrhage in the necrotic core (chevron) (Mallory’s trichrome); D) Speckled calcification (arrows)(H&E); E) Protruding calcium nodule (chevron) with two calcified plates (arrows)(H&E); F) Loose matrix (chevron) with adjacent dark blue dense matrix (star) (Mallory’s trichrome).

Supplementary Material 3. Supplement Table 1 Correlation of plaque composition with stenosis and percent wall volume (N = 186).

## Data Availability

No datasets were generated or analysed during the current study.

## Abbreviations

AS: Atherosclerosis
MRI: Magnetic resonance imaging
CEA: Carotid endarterectomy
LRNC: Lipid-rich necrotic core
LPH: Low-pressure hydrocephalus
IPH: Intraplaque hemorrhage
CI: Confidence interval
OR: Odds ratio
H&E: Hematoxylin and eosin
