# Supplementary figures and images for: Composition of carotid plaques differs between Chinese and US patients: a histology study
Source: Chin Neurosurg J. 2025 Oct 9;11:23. doi: 10.1186/s41016-025-00408-4 (PMC12509396; doi:10.1186/s41016-025-00408-4)

**Supplement Table 1**

**Correlation of plaque composition with stenosis and percent wall volume (N=186)**


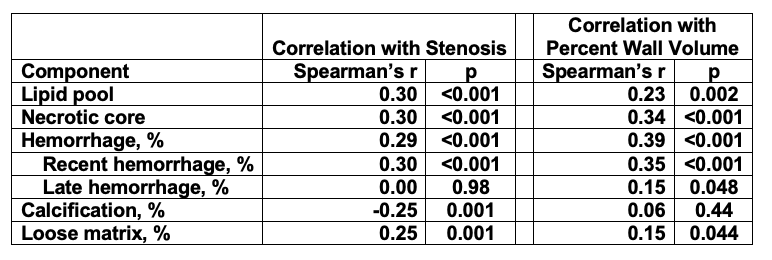

Supplement: Supplementary file 1 — Supplementary Material 1. Supplement Fig. 1 Left. Carotid plaque from a standard endarterectomy with disruption of the specimen. Right. Specimen from the modified surgical technique without disruption of the inner surface. Corresponding histologic sections are below. [file 41016_2025_408_MOESM1_ESM.docx]

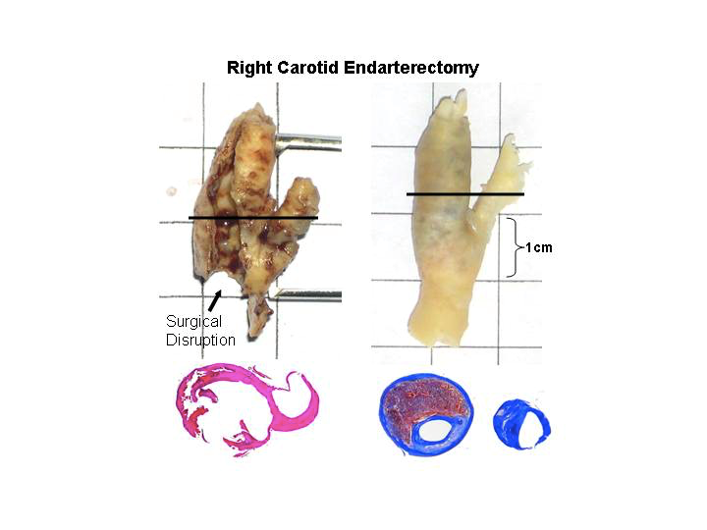

Supplement: Supplementary file 2 — Supplementary Material 2. Supplement Fig. 2 Carotid plaque components: A) Lipid pool (arrow)(H &E); B) Necrotic core with thick fibrous cap (arrow) (Mallory’s trichrome); C) Recent intraplaque hemorrhage into a necrotic core (star) (Mallory’s trichrome). Late intraplaque hemorrhage in the necrotic core (chevron) (Mallory’s trichrome); D) Speckled calcification (arrows)(H&E); E) Protruding calcium nodule (chevron) with two calcified plates (arrows)(H&E); F) Loose matrix (chevron) with adjacent dark blue dense matrix (star) (Mallory’s trichrome). [file 41016_2025_408_MOESM2_ESM.tif]

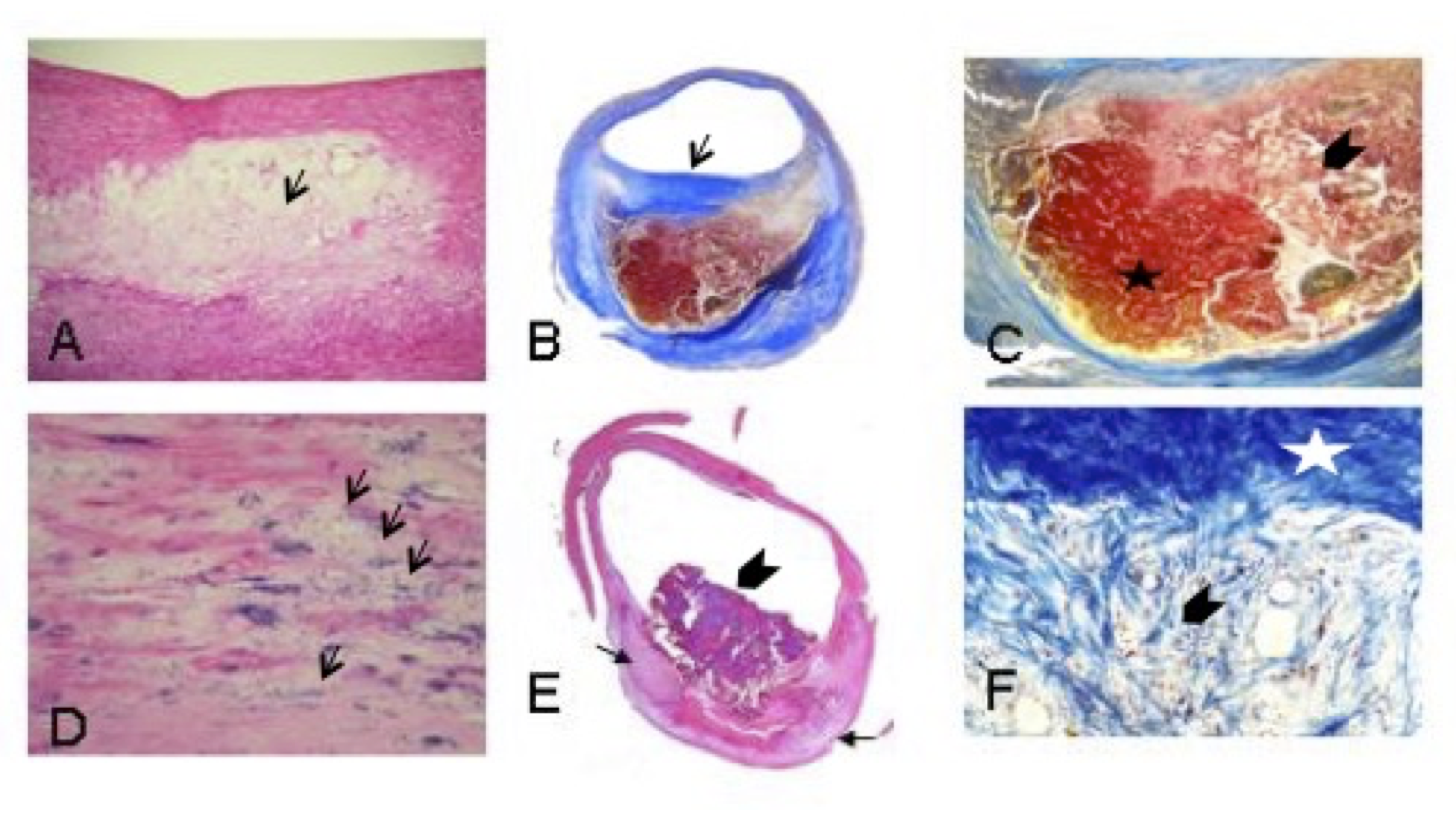

Supplement: Supplementary file 3 — Supplementary Material 3. Supplement Table 1 Correlation of plaque composition with stenosis and percent wall volume (N = 186). [file 41016_2025_408_MOESM3_ESM.tif]
